# Assessing the Clinical Utility of MRI for Preoperative Staging of Early-Stage Cervical Cancer in a Limited-Resource Setting: A Retrospective Cohort Study from Botswana

**DOI:** 10.21203/rs.3.rs-8041378/v1

**Published:** 2025-11-18

**Authors:** Elizabeth Corn, Kelly Becht, Bethel Adefres, Barati Monare, Rebecca Ketlametswe, Leatile Sedabadi, Juan Ariel Oliva Díaz, Peter Vuylsteke, Surbhi Grover, Lisa Bazzett-Matabele

**Affiliations:** Botswana-University of Pennsylvania Partnership; Yale University; Yale University; Botswana-University of Pennsylvania Partnership; Botswana-University of Pennsylvania Partnership; University of Botswana; Princess Marina Hospital; University of Botswana; Botswana-University of Pennsylvania Partnership; University of Botswana

**Keywords:** Cervical cancer, Botswana, magnetic resonance imaging (MRI), surgical staging, hysterectomy, limited-resource setting, gynecologic oncology

## Abstract

**Background:**

Cervical cancer is the number one cause of cancer-related mortality for women in Botswana, the care of which is complicated by the country’s severe shortage of gynecologic oncologists. A recent noninferiority trial suggests that some stage I cervical cancers can successfully be treated with simple hysterectomy (SH) instead of radical hysterectomy (RH), potentially easing the burden on specialists, reducing recovery time, and avoiding excess morbidity associated with RH. However, accurate risk assessment of invasion is crucial when choosing between SH and RH to ensure optimal patient outcomes.

**Methods:**

This study aims to investigate the feasibility of using magnetic resonance imaging (MRI) to stage early cervical cancer and guide surgical decision-making in a limited-resource setting in Botswana through a descriptive case series. Data were retrospectively collected for patients who underwent preoperative MRI and curative surgery for cervical cancer at Princess Marina Hospital (PMH) in Gaborone from September 2022 to December 2024. All patients were FIGO stage IA1-IB2 and had not received chemotherapy or radiation prior to surgery. Descriptive analysis comparing staging and tumor sizes across clinical diagnosis, MRI results, and final pathology was completed.

**Results:**

Thirty-two patients with early-stage cervical cancer were included in this study, 15 (47%) of whom underwent RH and 17 (53%) underwent SH. Staging between MRI and final surgical pathology was concordant in 16 (50%) patients. Four patients had the same stage across clinical, MRI, and surgical staging.

**Conclusions:**

The use of MRI for staging early-stage cervical cancer in Botswana may not accurately capture the extent of tumor invasion, highlighting the challenges of translating evidence for less invasive surgical strategies to resource-limited settings.

## Background

While cervical cancer accounts for less than 1% of cancer-related deaths in the United States, it is the fourth leading cause of cancer mortality among women globally (1, 2). As a largely preventable disease, the striking disparity in cervical cancer prevention and care between high-income countries (HICs) and low- and middle-income countries (LMICs) is particularly concerning. Patients in LMICs account for 86% of all cervical cancer cases of cervical cancer worldwide. This disparity is primarily driven by differences in vaccination against oncogenic human papillomavirus (HPV) variants and access to high-quality screening, which is widespread in high-resource regions but limited in LMICs (3, 4). In some regions of Africa, cervical cancer is the most common cause of cancer-related morbidity and mortality among women (5). Furthermore, the high prevalence of human immunodeficiency virus (HIV) in some countries in Africa contributes to an increased cervical cancer burden among patients living with HIV (6). It is estimated that Africa is operating at only 25% of its capacity to manage cervical cancer, highlighting substantial room for improving care delivery in LMICs (7).

Botswana faces significant challenges in the diagnosis and treatment of cervical cancer. Despite relatively strong healthcare infrastructure compared to other countries in the region, progress is hindered by a substantial shortage of healthcare personnel (8). Specifically, there remains a lack of gynecologic oncologists trained to manage gynecologic cancers and perform curative surgeries for early-stage cervical cancer. Until 2019, the country had no gynecologic oncologists, and currently, only one serves the entire nation. With 25% of patients in Botswana presenting with early-stage disease, patients face long surgical wait times and often receive suboptimal treatment (9). The standard of care for early-stage cervical cancer (FIGO stage IA2 - IB2) is a radical hysterectomy (RH), which involves the removal of parametrial tissues, the upper segment of the vagina, the uterus, and the cervix (10). While general gynecologic surgeons can perform simple hysterectomies (SH), complex cancer surgeries, including RH, are best performed by gynecologic oncologists, who are critically scarce in LMICs (9).

The standards of early cervical cancer treatment have been challenged by a recent phase 3 clinical trial, which has been the first to show that SH is noninferior to RH for patients with low-risk cervical cancer (11). Choosing SH for early-stage cervical cancer may reduce morbidity associated with injury to adjacent organs, pelvic nerves, and vasculature, which are intimately involved when performing RH (12). In LMICs, these findings may have even greater implications given the time, cost, and personnel constraints associated with performing complex surgeries like RH (11). One major limitation of the aforementioned clinical trial is the lack of inclusion of diverse populations, as all participants were from high-income countries (HICs). While LMICs would undoubtedly benefit the most from a shift towards SH for early cervical cancer, it remains unclear whether the conditions that contributed to the trial’s success would be present in settings such as Botswana.

Accurate staging of cervical cancer in HICs relies on multiple imaging modalities to provide comprehensive evaluation of tumor size, local invasion, and distant metastases. To account for variability in imaging technology availability in limited-resource countries, the 2018 FIGO guidelines were updated to accommodate the use of any imaging modality such as positron emission tomography/computed tomography (PET-CT), magnetic resonance imaging (MRI), ultrasound or computed tomography (CT) for staging, with accuracy dependent on the “skill of the operator” (13). This study aims to evaluate the accuracy of MRI in a limited-resource hospital for determining tumor size and assessing the likelihood of extra-cervical invasion.

## Methods

### Patient Population

This descriptive case series focuses on a cohort of patients diagnosed with early-stage cervical cancer who underwent MRI-based staging and curative surgery in a limited-resource setting in Botswana. All patients were referred to Princess Marina Hospital (PMH) gynecologic oncology multidisciplinary team (MDT) clinic in Gaborone, the capital city, and were clinically staged following excisional biopsies confirming cervical malignancy. MRIs were performed at Sir Ketumile Masire Teaching Hospital (SKMTH) to assess tumor size and spread, allowing for clinical staging and assessment of surgical candidacy. All patients underwent total abdominal hysterectomy (TAH) or RH and either unilateral or bilateral salpingo-oophorectomy or salpingectomy. Most patients also underwent pelvic lymph node dissection (PLND). Surgeries were performed by a gynecologic oncologist at PMH.

### Data Collection and Analysis:

Primary data for participants who underwent curative surgery for cervical cancer between September 2022 and December 2024 were collected from hospital medical records. Patients were excluded if they had only been imaged using CT, did not have available post-surgical pathology reports, or received chemotherapy or chemoradiation therapy (CRT) prior to surgery. Clinical staging was determined according to the 2018 International Federation of Gynecology and Obstetrics (FIGO) criteria, based on clinical examinations and radiographic imaging. Demographic variables, such as HIV status, marital status, and distance traveled to the hospital, were collected from the Botswana Prospective Cancer Cohort Research Electronic Data Capture (REDCap) database. Data collected from MRI reports included updates in FIGO staging based on MRI findings and measurements of enhancing cervical lesions. Actual tumor dimensions were collected from pathology reports of surgical specimens, along with the presence or absence of metastatic invasion of lymph nodes, parametrium, and vaginal margins. Staging was assigned in accordance with the 2018 FIGO criteria based on tumor size and narrative tumor descriptions in pathology reports. Due to the limited dataset, descriptive comparisons of staging and tumor sizing were performed across clinical diagnosis, MRI results, and final pathology.

## Results

### Patient Characteristics

Thirty-two patients met inclusion criteria and were included in this analysis. Patient ages at diagnosis ranged from 35 to 73 years, with a median age of 47.5 years (Table 1). Twenty-three patients (72%) were HIV-positive. Eight patients (25%) were married, and 17 (53%) had previously undergone cervical cancer screening. Most patients traveled from outside Gaborone to receive treatment at PMH, covering distances ranging from 10 km to 682 km (median 87 km). Most patients were not seen for their initial visit at PMH until several weeks after their biopsy at a referring institution, with the interval between visits ranging from 0 to 217 days. Six patients (19%) were not evaluated at PMH for more than three months following their initial biopsy. Most patients (75%) completed pelvic MRI within three weeks of their initial visit at PMH, with a median of 15 days between initial visit and MRI. One patient’s MRI preceded their diagnostic visit at PMH. Surgeries were performed a median of 68 days after MRI, with wait-times ranging from 27 days to 151 days.

### Clinical Diagnostics, MR Imaging, and Surgical Pathology

**Figure 1:**
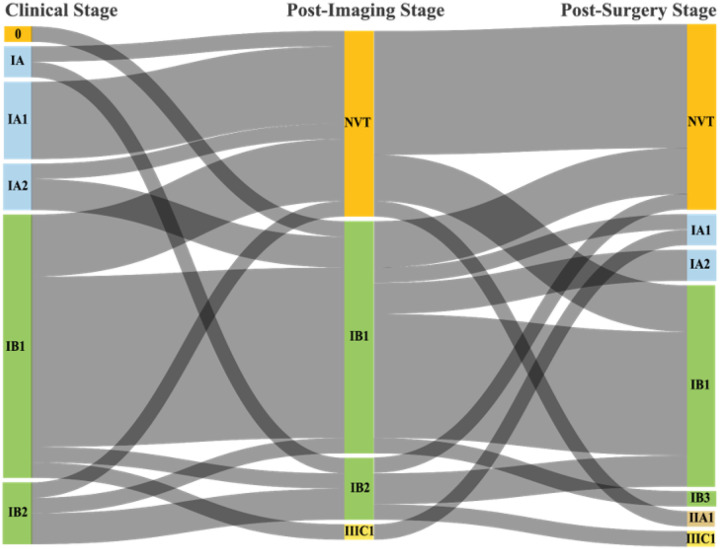
Clinical, MRI, Post-Surgical Stage Transitions Alluvial diagram showing stage transitions (clinical, post-imaging, and post-surgical) for 32 patients with early-stage cervical cancer. Flow widths are proportional to the number of patients transitioning between each stage. NVT, No visible tumor

Stages assigned by clinical exam prior to MRI ranged from stage 0 to IB2. All but one patient had MRI staging of IB2 or lower; the exception was a patient staged as IIIC1(r) due to diffusion restriction noted in the right common iliac chain lymph nodes on MRI (lymphatic invasion was not present following PLND and this patient’s final pathologic staging was IA1). Ten (30%) patients had no visible tumor on MRI, likely representing removal during prior excisional biopsy. Clinical staging was concordant between examination and MRI in 15 (47%) patients, and concordant between MRI and pathologic staging in 16 (50%) patients (Table 2). Individual patient stage transitions are illustrated in Fig. 1. Only four patients had concordant staging across clinical exam, MRI, and post-surgical assessments.

The surgical approach for each patient was determined by the on-site gynecologic oncologist in collaboration with the patient. Fifteen patients (47%) had a RH defined by removal of the uterus, cervix, parametria, 2 cm of the uterosacral ligaments, and upper 2 cm of the vagina. Seventeen (53%) patients had a TAH, constituting removal of the uterus and cervix. Twenty-six surgeries (81%) included PLND. On final pathology, only one of the 26 patients with PLND (4%) had a lymph node (1/11) positive for malignant invasion. One patient (3%), who underwent TAH, had positive vaginal margins on surgical pathology. Parametrial margins were negative for malignant invasion in all patients. Twelve patients (38%) had no visible tumor (NVT) on final surgical pathology. Absence of tumor on final pathology was accurately predicted in 7 of these patients with NVT on MRI.

## Discussion

The results of this study highlight the limitations of relying on clinical and radiological diagnostics, specifically with MRI, for surgical decision-making in resource-limited settings. In this group of patients treated in Gaborone, Botswana, MRI staging accurately reflected the final surgical stage in only half of those undergoing surgery for early-stage cervical cancer. Ten additional patients were downstaged following surgery. However, 19% of patients were underestimated by MRI, with final surgical evaluation revealing more advanced disease. This included one patient staged as IB2 on MRI and later found to have lymph node involvement, resulting in a final stage of IIIC1. Furthermore, one patient with vaginal cuff invasion on surgical pathology had previously been considered to have NVT on MRI.

While RH is considered the standard of care for cervical cancers staged IA2 to IB2, the added benefit of RH compared to SH is increasingly being questioned. RH carries greater intrinsic risks given the need to resect autonomic nerves associated with urinary and rectal function (14). Accidental injury to these nerves can lead to chronic complications such as urinary retention and incontinence (15). Surgeons opt for RH as a cautious approach to avoid the consequences of under-treating patients with potential malignant invasion of surrounding structures. The Simple Hysterectomy and Pelvic Node Assessment (SHAPE) trial was the first prospective study to demonstrate the noninferiority of SH to RH for cervical cancers staged IA1 to IB1. The study included patients classified as “low risk” based on preoperative LEEP biopsy and MRI staging according to specific criteria. Outcomes, defined by pelvic recurrences, were equivalent between the SH and RH groups. This trial raises the question of whether patients can be accurately stratified into a “low risk” group, and thus offered less radical surgery, especially in limited-resource settings (11).

At PMH in Botswana, the demand for gynecologic surgery far exceeds the available supply, which is constrained by factors such as limited hospital infrastructure and a shortage of well-trained gynecologic oncologists – a challenge common to many limited-resource settings. Performing RH as standard of care not only increases risks to the patient, but also maximizes time and resources required for each case. The ability to accurately identify “low-risk” patients who could safely undergo SH would free up resources, expanding access to treatment for a greater number of patients. Assessing the risk of invasion when deciding between surgery and CRT, as well as between SH and RH, is critical to minimizing harm (11). While recent evidence supports the efficacy of imaging techniques such as MRI and transvaginal ultrasound in staging cervical cancer, their success is likely not replicated to the same degree in limited-resource hospitals (16). This study suggests that the conclusions of the SHAPE trial may be more applicable to high-resource settings. Although less invasive surgery may offer greater benefits in limited-resource settings, our findings underscore the risks of relying solely on pre-surgical imaging to guide surgical decision-making.

Even when MRI suggests a “low-risk” tumor (e.g., < 2 cm, confined to cervix), the decision to perform less invasive surgery relies on confidence in imaging accuracy, which may be compromised by several factors. Firstly, MRI scanners in limited-resource settings may be older, have lower field strength, or lack advanced sequences important for assessing tumor margins (17, 18). Personnel challenges may also constrain the accuracy of MRI, as radiologists may have limited subspeciality training in gynecologic oncology (19, 20). Furthermore, high-resource centers often utilize standard imaging protocols and multidisciplinary tumor review boards to interpret radiologic findings in conjunction with clinical data. These systems are often less robust outside of academic institutions which may lead to increased variability in MRI interpretation. Radiological standardization in limited-resource settings is crucial; until improvements are made to increase the accuracy of imaging-based staging, the risks of performing less invasive oncologic surgeries may remain unacceptably high (21–23).

There are several limitations to this study. Data collection was complicated by heterogeneity in pathology reports, as FIGO 2018 stages were assigned by the researchers post-surgery based on information provided in each local pathology lab report. Significant variability among reports prevented precise comparisons of tumor dimensions between MRI and final pathology. The small sample size of this study additionally precluded the ability to statistically compare staging between cohorts. Furthermore, resource limitations and scheduling challenges resulted in large delays between MRI and surgery for some patients. It is unclear whether changes in tumor stage for individual patients reflect disease progression due to these delays rather than inaccuracy of MRI in predicting stage. Additionally, we are unable to comment on long-term outcomes of our cohort of patients due to follow-up challenges in a limited-resource setting. While this study highlights cervical cancer staging capabilities in a specific limited-resource setting, broader research across health systems is needed to better understand the regional landscape of cancer care to guide quality improvements that enhance patient outcomes.

## Conclusion

This study highlights that MRI-based assessment of staging for early-stage cervical cancer in Botswana may not reliably capture tumor size or extent of invasion, raising concerns about its use in guiding surgical decision-making in limited-resource settings. As the field of gynecologic oncology moves towards more favorable, less invasive treatment strategies, it is essential to understand that patients in LMICs – those who stand to benefit the most from such advancements – are often left out of the equation. Strengthening diagnostic infrastructure in LMICs is a crucial step towards ensuring equitable access to cervical cancer care and improving outcomes worldwide.

## Figures and Tables

**Table 1:** Demographics of surgical candidates with early-stage cervical cancer diagnosis

Summarized demographic data	Median (IQR) [Range]	Number (%)
**Age at diagnosis**	47.5 (13) [35–73]	
**Distance traveled to PMH (km)**	87 (270.5) [10–682]	
**Days between biopsy and diagnostic visit**	55 (32.5) [0–217]	
**Days between diagnostic visit and MRI**	15 (11.5) [2–64]	
**Days between MRI and surgery**	68 (49.5) [27–151]	
**HIV positive**		23 (71.9%)
**Married**		8 (25%)
**Previously screened for cervical cancer**		17 (53.1%)

PMH, Princess Marina Hospital; MRI, magnetic resonance imaging; HIV, human immunodeficiency virus; IQR, interquartile range.

**Table 2:** Distribution of clinical, post-imaging, and post-surgical stage changes

	Number (n = 32)	Percent
**Clinical stage vs. MRI stage**		
**Upstaged**	6	19%
**Downstaged**	1	3%
**None**	15	47%
**NVT on MRI**	10	31%
**MRI stage vs. pathology stage**		
**Upstaged**	6	19%
**Downstaged**	10	31%
**None**	16	50%

MRI, magnetic resonance imaging; NVT, no visible tumor

## Data Availability

The datasets used and/or analysed during the current study are available from the corresponding author on reasonable request

## Abbreviations

HIC: high income country
LMICs: low-and middle-income countries
HPV: human papilloma virus
HIV: human immunodeficiency virus
FIGO: International Federation of Gynaecology and Obstetrics
RH: radical hysterectomy
SH: simple hysterectomy
PET-CT: positron emission tomography/computed tomography
PMH: Princess Marina Hospital
MDT: multidisciplinary team
SKMTH: Sir Ketumile Masire Teaching Hospital
TAH: total abdominal hysterectomy
PLND: peritoneal lymph node dissection
CRT: chemoradiation therapy
NVT: no visible tumor
